# Comparative Evaluation of Sentinel Lymph Node Detection Rates in Breast Cancer Surgery: “ICG + Patent Blue” Versus “99mTc + Patent Blue”, a 11-Year Single-Center Study

**DOI:** 10.3390/cancers18060959

**Published:** 2026-03-16

**Authors:** Ines Hfaiedh, Arrigo Fruscalzo, Joy Shannon Sudan, Anis Feki, Benedetta Guani

**Affiliations:** 1 University Hospital of Fribourg, Hôpital Fribourgeois (HFR), 1752 Fribourg, Switzerland; arrigo.fruscalzo@h-fr.ch (A.F.); anis.feki@h-fr.ch (A.F.); benedetta.guani@h-fr.ch (B.G.); 2 Department of Medicine, Faculty of Science and Medicine, University of Fribourg, 1700 Fribourg, Switzerland; joy.sudan@hotmail.ch

**Keywords:** breast cancer, sentinel lymph node biopsy, dual-tracer technique, indocyanine green, technetium-99m, patent blue dye, detection failure

## Abstract

**Background**: Breast cancer is the most common malignancy in women, and sentinel lymph node (SLN) biopsy is essential for accurate nodal staging while avoiding unnecessary axillary dissection. **Aim**: This study aimed to compare SLN detection rates between two dual-tracer techniques: indocyanine green plus patent blue (ICG + PB) and technetium-99m plus patent blue (99mTc + PB), and to identify factors associated with detection failure for each tracer. **Methods**: All clinically node-negative breast cancer patients undergoing SLN biopsy between January 2014 and December 2024 were retrospectively evaluated. SLN detection was considered successful when at least one node was identified intraoperatively and confirmed histologically. Multivariate analysis assessed clinical and tumor-related predictors of failure. **Results**: A total of 269 procedures (258 patients) were analyzed, including 152 ICG + PB and 117 99mTc + PB procedures. Detection rates were comparable between groups (95.4% vs. 94.9%, *p* = 0.96), with no significant differences in the number of SLNs retrieved or nodal positivity. Multivariate analysis identified increasing patient age as the only independent predictor of PB failure, while no variables were associated with ICG failure. Tumor location in the upper-inner quadrant was the sole predictor of 99mTc failure. **Conclusions**: ICG + PB and 99mTc + PB provide equivalent and high SLN detection rates. ICG appears to be a robust, radiation-free alternative with no identifiable predictors of failure, supporting its role as an effective mapping strategy, particularly in centers aiming to optimize workflow and patient safety, despite the limited available data on its efficacy.

## Simple Summary

Breast cancer is the most common cancer affecting women worldwide. To determine whether cancer has spread, surgeons often perform a sentinel lymph node (SLN) biopsy, which identifies the first lymph node(s) likely to contain cancer cells while avoiding more extensive lymph node removal. This study compared two dual-tracer techniques used to detect these lymph nodes: indocyanine green combined with patent blue dye (ICG + PB) and technetium-99m combined with patent blue dye (99mTc + PB). We found that both methods had similarly high detection rates. Increasing age was associated with the failure of patent blue detection, and tumors located in the upper-inner quadrant were linked to technetium-99m detection failure. No factors were associated with failure when using indocyanine green. These findings suggest that indocyanine green is a reliable, radiation-free alternative for SLN mapping in breast cancer surgery.

## 1. Introduction

Breast cancer remains the most frequently diagnosed malignancy among women worldwide and accounts for approximately 1% of all breast cancer cases in men. The sentinel lymph node (SLN), defined as the first lymph node receiving lymphatic drainage from the primary tumor, represents the initial site of potential metastatic spread.

SLN mapping has substantially contributed to therapeutic de-escalation in breast cancer management by reducing the need for routine axillary lymph node dissection and improving postoperative morbidity and quality of life. Moreover, SLN biopsy is essential for accurate staging: in the absence of SLN metastasis, non-sentinel lymph nodes are negative in approximately 99% of cases, allowing for reliable nodal assessment while avoiding overtreatment [1].

The current gold standard techniques for SLN mapping rely on the use of radioisotopes, most commonly technetium-99m (99mTc), often combined with blue dye, such as Patent Blue (PB), to enhance intraoperative detection. While this dual-tracer approach achieves high detection rates, it presents several limitations, including exposure to ionizing radiation, dependence on nuclear medicine facilities, logistical constraints, regulatory requirements, and increased costs. Blue dye alone, although inexpensive and widely available, is associated with lower detection rates and carries a risk of allergic reactions.

Indocyanine Green (ICG) fluorescence imaging has emerged as a promising alternative or adjunct to conventional tracers. ICG offers real-time visualization of lymphatic pathways, does not involve radiation exposure, and may facilitate intraoperative identification of SLNs, particularly in minimally invasive and resource-limited settings. Additional theoretical advantages include improved visualization in obese patients and the potential to shorten operative times and streamline surgical workflow. However, data regarding predictors of detection failure and comparative performance with standard dual-tracer techniques remain limited and heterogeneous.

The primary objective is to compare the SLN detection failure rates between two dual-tracer techniques:

Group 1: Indocyanine Green (ICG) + Patent Blue (PB).

Group 2: Technetium-99 (99mTc) + Patent Blue (PB).

The secondary objective is to identify clinical or tumor-related factors associated with detection failure for each tracer (PB, ICG, 99mTc).

## 2. Materials and Methods

This retrospective single-center study was conducted in the Department of Gynecology and Obstetrics of HFR Fribourg, including all procedures of SLN biopsy in patients with clinically node-negative (cN0) breast cancer between 1 January 2014 and 31 December 2024.

Eligible patients underwent SLN mapping using one of two dual-tracer protocols: ICG + PB or 99mTc + PB.

Data were collected from medical files, operative reports, and final pathological reports.

Two types of failure were defined:

- Technical failure: absence of intraoperative SLN detection by both tracers.
- Histological failure: absence of identifiable lymph node tissue on final pathology despite presumed intraoperative SLN detection.

Successful detection was defined as the identification of ≥1 SLN with at least one tracer and confirmation of lymph node tissue on pathology.

Descriptive statistics were used to summarize patient, tumor, and procedural characteristics. Continuous variables were expressed as medians with interquartile ranges (IQRs) and compared using the Mann–Whitney U test, due to non-normal distribution. Categorical variables were reported as frequencies and percentages and compared using the Chi-square test or Fisher’s exact test, as appropriate.

Sentinel lymph node detection failure was analyzed as a binary outcome. Multivariate logistic regression models were constructed to identify independent clinical and tumor-related predictors of detection failure for each tracer modality (PB, ICG, and 99mTc). Variables with clinical relevance and those showing potential association in univariate analysis were entered into the multivariate models. Results were reported as adjusted odds ratios (ORs) with 95% confidence intervals (CIs).

All tests were two-sided, and a *p*-value < 0.05 was considered statistically significant. Statistical analyses were performed using Microsoft Excel and Stata 19.0.

Inclusion criteria included the following:

- Adult patients with biopsy-proven breast cancer, negative axilla on clinical and/or imaging assessment, eligible for SLN biopsy at HFR.
- Documented consent for research data use.

Exclusion criteria were as follows:

- Patients under 18 years old;
- Planned axillary lymph node lymphadenectomy;
- Previous axillary surgery;
- Clinically or radiologically node-positive disease;
- Refusal or absence of consent for the research use of data.

## 3. Results

A total of 258 patients were included (250 women, 8 men). Median age was 62 years (51–71), ranging from 27 to 85 years. Female-to-male ratio was 32.6:1.

Median BMI was 26.04 kg/m^2^ (23.05–29.97), ranging from 16.88 to 48.00. Weight categories were distributed as follows: 3% underweight, 40% normal weight, 32% overweight, 19.3% class I obesity, 2.2% class II obesity, and 3.3% class III obesity.

Median gravidity and parity among women were both 2 (1–3). Family history of breast cancer was reported in 90 cases (33.5%). Previous breast or axillary surgery was documented in 15 cases (5.6%). Menopausal status distribution was 69% postmenopausal without HRT, 2% menopausal with HRT, 23% premenopausal, and 6% perimenopausal.

In total, 269 SLN procedures were analyzed: 239 unilateral and 11 bilateral in women (22 procedures), and 8 unilateral in men.

**Figure 1:**
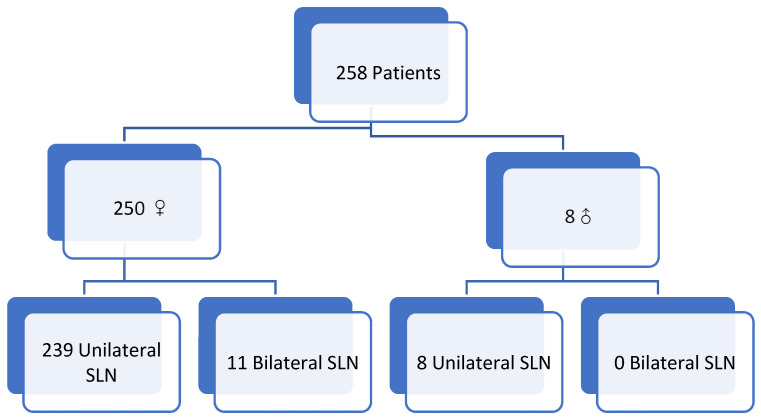
Flow chart of collected cases.

The cohort was divided into two groups: (Figure 1).

- Group 1 (ICG + PB): 152 procedures.
- Group 2 (99mTc + PB): 117 procedures.

**Figure 2:**
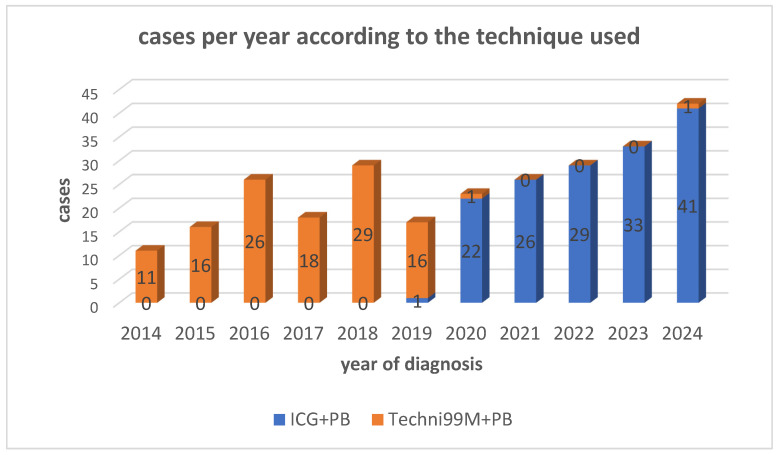
Distribution of diagnosed cases per year according to the technique used.

The distribution of diagnosed cases per year according to the technique used is illustrated in Figure 2; in fact, after 2019, sentinel lymph node mapping was routinely performed using ICG + PB following a departmental protocol change. A single 99mTc + PB guided procedure was conducted in 2020 during the transition period, and another isolated case occurred in 2024 due to particular logistical considerations. These two cases account for the limited use of technetium after protocol standardization.

1. Group Comparability

Concerning the epidemiologic characteristics, the two groups were comparable regarding sex distribution, BMI, family history of breast cancer or other malignancies, gravidity, parity, and menopausal status (Table 1); however, patients in Group 2 were slightly older than those in Group 1 (median age 66 (IQR 53–72) vs. 61 (IQR 49–70), *p* = 0.03), and prior ipsilateral breast surgery was more frequent in Group 2 (9.4% vs. 2.6%, *p* = 0.02) (Table 1). Regarding tumor clinical characteristics, no significant differences were observed in tumor laterality, focality (unifocal, bifocal, or multifocal), number of tumor foci, or tumor size, although tumors located in the lower outer quadrant were more frequent in Group 1 compared with Group 2 (22.4% vs. 12.8%, *p* = 0.02) (Table 2). TNM staging did not differ significantly between the two groups (Table 3). Similarly, histopathological features were comparable in terms of histologic subtype, presence of ductal carcinoma in situ, Ki-67 proliferation index, and histologic grade; estrogen receptor expression showed no significant difference. In contrast, progesterone receptor positivity was slightly higher in Group 1 than in Group 2 (73% vs. 69%, *p* = 0.02), and HER2-positive tumors were significantly more frequent in Group 1 (18.4% vs. 4.6%, *p* < 0.001), probably due to an increased use of neoadjuvant chemotherapy in this subtype. (Table 4). With regard to perioperative and pathological outcomes, there were no significant differences in the total number of sentinel lymph nodes retrieved. However, the number of nodes classified as “sentinel” or “accessory” varied according to the surgeon’s designation in the final operative report. Concordance between intraoperative surgical assessment and final pathological findings, or rates of sentinel lymph node positivity (micro- or macrometastases); however, no intraoperative frozen-section analysis was performed in Group 1, in accordance with institutional protocol changes (Table 5).

2. Comparison of Detection Failure Rates (Table 6)

Sentinel lymph node detection rates were comparable between the two groups. In Group 1 (ICG + patent blue), the detection rate was 95.4% (144/152), while in Group 2 (99mTc + patent blue), it reached 94.9% (111/117). No statistically significant difference was observed between the detection strategies (*p* = 0.96).

**Figure 3:**
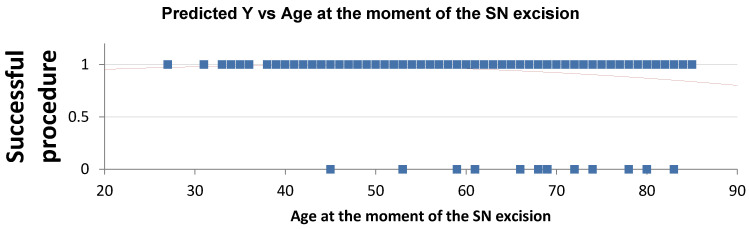
Global failure rates increasing with advancing age. Blue dots represent individual patient outcomes (1 = successful procedure, 0 = failure). The red line represents the predicted probability of successful detection derived from the logistic regression model.

**Figure 4:**
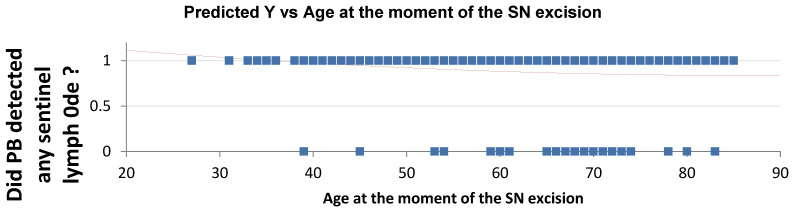
PB-specific failure increased with advancing age. Blue dots represent individual patient outcomes (1 = successful PB detection, 0 = failure of PB detection). The red line represents the predicted probability of successful PB detection derived from the logistic regression model.

3. Factors Associated with Detection Failure by Tracer:

Multivariate analysis evaluated potential predictors of sentinel lymph node detection failure, including age, BMI, family history of breast cancer, prior ipsilateral breast surgery, tumor location, tumor size, and nodal status (N+). All 269 procedures included the use of Patent Blue; in this subgroup, increasing age was identified as the only independent predictor of PB detection failure (Table 7), with both overall detection failure and PB-specific failure rates rising progressively with advancing age (Figure 3 and Figure 4). In contrast, no clinical or tumor-related variable was significantly associated with ICG detection failure (Table 8). Regarding 99mTc, tumor location in the lower inner quadrant (LIQ) was the only variable significantly associated with detection failure (*p* = 0.01) (Table 9).

## 4. Strengths and Limitations of the Study

Key strengths of this study include the large sample size, the extended 11-year inclusion period, and the high level of procedural standardization, including uniform tracer dosing and consistent surgical technique within a single experienced team. These factors reduce technical heterogeneity and enhance internal validity.

However, the retrospective and non-randomized design, together with the single-center setting, may limit external validity and preclude definitive causal conclusions. In addition, analyses were conducted at the procedure level, although a subset of patients underwent bilateral procedures, introducing potential non-independence of observations. This may have led to an underestimation of variance and overstatement of statistical precision in the absence of clustering or robust variance adjustment.

A further limitation is the relatively low number of detection failure events observed in both groups, which may reduce the statistical power to detect weak but clinically relevant associations. Consequently, the absence of significant predictors of ICG detection failure should be interpreted with caution. Further studies with larger sample sizes or multicenter designs would be valuable to better explore potential predictors of detection failure.

Finally, despite standardized protocols, the operator’s surgical learning curve and residual inter-operator variability may have influenced detection outcomes.

## 5. Discussion

### 5.1. Comparison of Dual-Tracer Techniques

According to our results, ICG with PB and 99mTc with PB provide equivalent sentinel lymph node detection rates in breast cancer, with both methods achieving high identification rates and comparable clinical effectiveness.

Recent prospective trials and systematic studies, including substantial patient cohorts, have evaluated dual-tracer approaches for sentinel lymph node (SLN) mapping in early breast cancer. Nguyen et al. analyzed approximately 150 patients per group and reported no failed mappings with either ICG–radioisotope (ICG-RI) or blue dye–radioisotope (BD-RI) combinations, corresponding to a 100% SLN identification rate in both arms. Similarly, Da Silva Sa et al. demonstrated a 100% detection rate with the combined use of indocyanine green (ICG) and patent blue, compared with 93.9% for ICG alone and 78.8% for patent blue alone. In a cohort of 117 patients, Kolbow et al. reported an overall identification rate of 93.2% using a dual radioactive and fluorescent technique. Furthermore, in the large multicenter SENTINA trial conducted by Kuehn et al., which included 1022 patients undergoing upfront SLN biopsy, the detection rate reached 99.1% (95% CI 98.3–99.6). These findings collectively indicate that dual-tracer strategies, particularly those combining fluorescence or radioisotopes with blue dye, consistently achieve identification rates exceeding 95% in early-stage breast cancer. However, detection rates may be significantly lower in the post-neoadjuvant setting, underscoring the importance of clinical context when interpreting these results [2,3,4,5].

Comparative studies confirm that ICG-based techniques are noninferior to the gold standard 99mTc plus blue dye, with similar sensitivity for metastatic node detection and no significant difference in the number of SLNs identified or failed mapping rates [2,3,4,6]. Both methods reliably detect macrometastatic and micrometastatic nodes, and the median number of SLNs retrieved per procedure is comparable (typically 2–3 nodes) [2,4,5].

ICG offers additional advantages, including a lower risk of allergic reactions and skin tattooing compared to blue dye, and may streamline workflow by eliminating the need for preoperative lymphoscintigraphy, though it requires specialized imaging equipment and may incur higher upfront costs [2,3,7]. In fact, ICG eliminates the need for nuclear medicine infrastructure, preoperative lymphoscintigraphy, and handling of radioactive materials, which is especially advantageous in centers without access to nuclear medicine facilities or where workflow optimization is a priority [7,8,9]. This streamlines scheduling, reduces waiting times, and increases flexibility for surgical teams [7]. Economic analyses show that ICG reduces costs by simplifying the care pathway and avoiding hospitalization for radiotracer injection [7,10]. The safety profile of indocyanine green (ICG) is excellent, as demonstrated by large clinical series and systematic reviews. The incidence of severe adverse reactions, including anaphylaxis, is consistently reported to be below 0.05%. The most common clinically significant adverse events are hypersensitivity reactions, such as urticaria and anaphylaxis, although these remain rare. The FDA labeling reports rare fatal cases of anaphylaxis, highlighting the need for appropriate monitoring and resuscitation preparedness, particularly in patients with known iodide allergy. ICG is not contraindicated in pregnancy, as transplacental passage is minimal and no evidence of maternal or fetal harm has been demonstrated [2,11,12,13,14,15,16].

Meta-analyses confirm that ICG is superior to blue dye alone and equivalent to radiocolloid tracers, and that dual-tracer methods maximize SLN identification and minimize false-negative rates. Pooled data report overall SLN detection rates exceeding 95–98% with ICG, significantly higher than blue dye alone, and comparable to technetium-based radiocolloids. In large prospective studies such as the SENTINA trial, detection rates reached 99.1% before neoadjuvant chemotherapy but decreased to 80.1% after chemotherapy in initially node-positive patients, with a false-negative rate of 14.2%, particularly when only one sentinel node was retrieved. These findings support the use of dual-tracer techniques to optimize identification and reduce false-negative results, especially in the neoadjuvant setting [5,17]. The current consensus supports the use of either dual-tracer method for optimal SLN detection in breast cancer, with selection based on institutional resources, workflow, and patient safety considerations.

### 5.2. Predictive Factors for Detection Failure

SLN detection in breast cancer surgery is multifactorial and varies by tracer modality. ICG, 99mTc, and PB each have distinct profiles of risk factors for detection failure.

#### 5.2.1. Indocyanine Green (ICG)

ICG fluorescence imaging demonstrates high SLN detection rates, often exceeding 90% [2,4,18,19].

In our study, we found that ICG performance was not affected by patient or tumor characteristics, reinforcing its reliability across diverse clinical scenarios. However, in the literature, we found that failure can occur in patients with elevated body mass index (BMI), as increased adiposity impairs near-infrared signal penetration and lymphatic migration [20,21]. Macrometastatic involvement of SLNs also reduces ICG detection rates, likely due to lymphatic obstruction [21]. Technical factors such as suboptimal injection technique and insufficient ICG dose may further contribute to failure [20]. Despite these limitations, ICG is consistently superior to patent blue and at least equivalent to 99mTc in SLN identification [2,3,13,20].

#### 5.2.2. Technetium-99 (99mTc)

99mTc-based lymphoscintigraphy is the gold standard for SLN mapping. Based on our findings, failure rates increase with LIQ tumors’ localization, a finding that may relate to lymphatic drainage patterns or technical aspects of isotope migration, which aligns with the published literature; in fact, medially or centrally located tumors are associated with failure [8].

Other factors found according to published data are age (≥70 years), high BMI (≥30 kg/m^2^), and nonpalpable tumors [8,17,22,23,24]. Extensive nodal involvement (≥10 positive nodes) and prior mantle field radiation are additional risk factors [8,23].

Technical aspects, such as deep injection (peritumoral/intratumoral) and radiotracer uptake in internal mammary nodes, also reduce detection rates [17,25,26].

Surgeon experience is a modifiable factor, with less experienced operators having higher failure rates [11,24].

#### 5.2.3. Patent Blue (PB)

PB alone is associated with the highest SLN detection failure rates among the three tracers [2,4,11,13,27]. Our study demonstrates that increasing age emerged as the sole independent predictor of PB failure, possibly reflecting age-related changes in lymphatic architecture.

Published data show that increasing age, high BMI, and medial or lower inner quadrant tumor location are independent predictors of failure [11,17,24]. Blue dye is also more susceptible to technical errors, such as inadequate injection or rapid diffusion, and is inferior to both ICG and 99mTc in terms of sensitivity and false-negative rates [3,4,13,27]. Combination techniques (blue dye plus 99mTc or ICG) significantly improve detection rates and mitigate individual tracer limitations [2,4,13,27].

In summary, dual-tracer techniques using indocyanine green (ICG) plus patent blue and technetium-99m plus patent blue achieved similarly high sentinel lymph node (SLN) detection rates, consistent with published data supporting the non-inferiority of ICG to radiocolloid methods and its superiority to blue dye alone. Detection failure was tracer-specific and multifactorial. While ICG performance in our cohort was unaffected by clinical variables, 99mTc failure was associated with tumor location and technical factors, and increasing age was the only independent predictor of patent blue failure. Overall, dual-tracer approaches remain the most reliable strategy and continue to be recommended in many guidelines. The choice between ICG and 99mTc as monotherapy should be guided by institutional resources, patient characteristics, and logistical considerations. Currently, there is no conclusive evidence supporting the systematic use of one tracer over the other in all clinical scenarios; both are acceptable options, while dual-tracer mapping is preferable whenever feasible [28,29,30].

## 6. Conclusions

In this 11-year single-center cohort, the dual-tracer techniques ICG + PB and 99mTc + PB demonstrated equivalent sentinel lymph node detection rates, supporting the use of ICG as a safe and effective alternative to radioisotopes.

Age was identified as the only significant risk factor for PB failure, while ICG showed no identifiable predictors of detection failure; however, the low number of failure events limits statistical power and precludes definitive conclusions.

The strong performance of ICG across all patient and tumor profiles positions it as a robust, radiation-free option for SLN mapping, particularly valuable in centers without nuclear medicine facilities or seeking to optimize workflow and safety.

## Figures and Tables

**Table 1:** Comparative analysis of the groups regarding epidemiologic characteristics.

Variable N (%)		Group 1: ICG + PB (*n* = 152)	Group 2: 99mTc + PB (*n* = 117)	*p* Value
Age (years)		61 (49–70)	66 (53–72)	0.03
Sex	Female	147 (96.7%)	114 (97.4%)	0.73
	male	5 (3.3%)	3 (2.6%)	
BMI (Kg/m^2^)		26 (23–30)	26.22 (23–30)	0.92
Family history of breast cancer		52 (34.21)	38 (32.48)	0.77
History of homolateral breast surgery		4 (2.63%)	11 (9.40%)	0.02
Other familial cancers		54 (33.5%)	37 (31.6%)	0.50
Gravidity		2 (1–3)	2 (1–3)	0.67
Parity		2 (1–3)	2 (1–3)	0.11
Menopausal status	Pre-menopausal	41 (27%)	20 (17%)	0.14
	Peri-menopausal	5 (3.4%)	10 (8.7%)	0.17
	Post-menopausal without HRT	100 (68%)	81 (71%)	0.82
	Menopausal with HRT	1 (0.6%)	3 (2.6%)	0.42

**Table 2:** Comparative analysis of the groups regarding tumor clinical characteristics.

Variable N (%)		Group 1: ICG + PB (*n* = 152)	Group 2: 99mTc + PB (*n* = 117)	*p* Value
Right side		73 (48%)	59 (50.4%)	0.7
Left side		79 (52%)	58 (49.6%)	
Bilateral		13 (8.6%)	18 (15.4%)	0.08
Focality	Unifocal	127 (83.6)	98 (83.8%)	0.96
	Bifocal	12 (7.9%)	12 (10.3%)	0.5
	Multifocal	13 (8.6%)	7 (6%)	0.43
Quadrant distribution	UQQ	75 (49.3%)	52 (44.4%)	0.43
	UIQ	30 (19.7%)	27 (23.1%)	0.51
	LOQ	34 (22.4%)	15 (12.8%)	0.02
	LIQ	19 (12.5%)	21 (17.9%)	0.21
	Retroareolar area	12 (7.9%)	14 (12%)	0.26
Multiple localization		20 (13.2%)	14 (12%)	0.77
Number of tumors		1 (1–1)	1 (1–1)	0.22
Size of the tumor		16 (10–23)	15 (9–23)	0.12

**Table 3:** Comparative analysis of the groups regarding TNM classification.

Variable N (%)		Group 1: ICG + PB (*n* = 152)	Group 2: 99mTc + PB (*n* = 117)	*p* Value
T	Tx	2 (1.3%)	1 (0.9%)	0.71
	Tis	16 (10.5%)	10 (8.5%)	0.72
	T1	102 (67.1%)	82 (70%)	0.99
	T2	32 (21.1%)	30 (25.6%)	0.55
	T3	7 (4.6%)	4 (3.4%)	0.76
	T4	4 (2.6%)	1 (0.9%)	0.39
	The advanced T stage	1 (1–2)	1 (1–2)	0.92
N	Nx	4 (2.6%)	0 (0%)	0.08
	pN0	115 (75.7%)	89 (76%)	0.94
	pN1	32 (21%)	24 (20.6%)	0.91
	pN2	1 (0.7%)	4 (3.4%)	0.10
M	Mx	100 (65.8%)	85 (72.6%)	0.23
	M0	52 (34.2%)	32 (27.4%)	

**Table 4:** Comparative analysis of the groups regarding the histopathological profile of the tumor.

Variable N (%)		Group 1: ICG + PB (*n* = 152)	Group 2: 99mTc + PB (*n* = 117)	*p* Value
Histological type	NST	97 (63.8%)	82 (70.1%)	0.28
	DCIS	8 (5.3%)	7 (6%)	0.78
	Lobular	21 (13.8%)	13 (11.1%)	0.51
	Rare tumors	26 (17.1%)	15 (12.8%)	0.33
Associated DCIS		85 (57.4%)	64 (55.7%)	0.06
Hormonal receptors	ER (+)	128 (84.2%)	98 (86.7%)	0.60
	Pr (+)	111 (73%)	78 (69%)	0.02
	HER2 (+)	28 (18.4%)	5 (4.6%)	*p* < 0.001
	Ki67(%)	15 (10–30)	15 [7.75–28.75]	0.77
Grade	1	19 (12.6%)	29 (24.8%)	0.07
	2	100 (66.2%)	62 (53%)	0.10
	3	31 (20.5%)	26 (22.2%)	0.47

**Table 5:** Comparative analysis of the groups regarding perioperative and pathology data.

Variable N (%)		Group 1: ICG + PB (*n* = 152)	Group 2: 99mTc + PB *(n* = 117)	*p* Value
Intraoperative frozen section n (%)		0	60 (51.3)	<0.001
Number of lymph nodes	Total	3 (2–4)	3 (2–4)	0.07
	Sentinel	2 (1–2)	1 (1–2)	<0.001
	Accessory	1 (0–1)	1 (1–3)	<0.001
	Discordance between surgeon and pathologist	37 (24.3%)	26 (22.2%)	0.69
	Overestimation	12 (32.4%)	5 (19.2%)	0.23
	Underestimation	25 (67.6%)	21 (80.8%)	0.23
N (+)	Yes	28 (18.4%)	24 (20.5%)	0.68
	Micrometastasis	10 (35.7%)	13 (54.2%)	0.19
	Macrometastasis	19 (67.9%)	13 (54.2%)	0.41

**Table 6:** Comparative analysis of the groups regarding success/failure.

Groups	Success	Failure	*p* Value
G1 (BP + ICG) n = 152	144 (95.4%)	8 (4.6%)	0.96
G2 (BP + 99mTc) n = 117	111 (94.9%)	6 (5.1%)	

**Table 7:** Multivariate analysis of factors predicting PB mapping failure.

Predictors		*p*-Value	OR	IC 95%
Age		0.04	0.96	0.93–0.99
BMI		0.62	0.97	0.90–1.05
Family history of breast cancer		0.98	1.07	0.44–2.61
History of ipsilateral breast surgery		0.29	0.55	0.13–2.29
Tumor location	UOQ	0.17	0.40	0.13–1.23
	UIQ	0.26	2.74	0.58–12.94
	LOQ	0.45	0.67	0.25–1.80
	LIQ	0.07	0.25	0.07–0.96
	Retroareolar area	0.85	1.00	0.21–5.05
	Multiple	0.61	1.32	0.27–6.46
Tumor size		0.95	1.00	0.97–1.03
Metastatic SLN (all stages not N0)		0.97	0.92	0.33–2.54

**Table 8:** Multivariate analysis of factors predicting ICG mapping failure.

Predictors		*p*-Value	OR	IC 95%
Age		0.24	0.96	0.90–1.08
BMI		0.65	1.01	0.86–1.02
Family history of breast cancer		0.58	4.85	0.52–44.84
History of ipsilateral breast surgery		0.21	0.84	0.04–16.99
Tumor location	UOQ	0.93	2.89	0.20–41.57
	UIQ	0.14	5.15	0.27–98.03
	LOQ	0.83	2.80	0.23–33.81
	LIQ	0.96	2.42	0.14–41.90
	Retroareolar area	0.28	7.37	0.14–396.46
	Multiple	0.22	2.39	0.12–49.11
Tumor size		0.83	1.02	0.96–1.08
Metastatic SLN (all stages not N0)		0.31	2.93	0.36–28.46

**Table 9:** Multivariate analysis of factors predicting 99mTc mapping failure.

Predictors		*p*-Value	OR	IC 95%
Age		0.51	1.01	0.95–1.08
BMI		0.49	0.97	0.85–1.09
Family history of breast cancer		0.38	0.48	0.11–1.99
History of ipsilateral breast surgery		0.18	0.35	0.06–2.21
Tumor location	UOQ	0.08	0.22	0.03–1.68
	UIQ	0.71	3.70	0.25–54.44
	LOQ	0.60	3.29	0.16–67.99
	LIQ	0.01	0.09	0.01–0.89
	Retroareolar area	0.08	0.18	0.02–1.49
	Multiple	0.30	0.38	0.03–4.33
Tumor size		0.57	0.99	0.95–1.05
Metastatic SLN (all stages not N0)		0.44	0.61	0.11–3.34

## Data Availability

The data presented in this study are available on reasonable request from the corresponding author. The data are not publicly available due to institutional and ethical restrictions.

## Abbreviations

The following abbreviations are used in this manuscript:

SLN	Sentinel Lymph Node
SLNB	Sentinel Lymph Node Biopsy
ICG	Indocyanine Green
PB	Patent Blue
99mTc	Technetium-99m
BMI	Body Mass Index
DCIS	Ductal Carcinoma In Situ
HRT	Hormone Replacement Therapy
TNM	Tumor–Node–Metastasis (staging system)
UQQ	Upper-outer Quadrant
UIQ	Upper-inner Quadrant
LOQ	Lower-outer Quadrant
LIQ	Lower-inner Quadrant
